# Impact of liver graft steatosis on long-term post-transplant hepatic steatosis and fibrosis via magnetic resonance quantification

**DOI:** 10.3389/fmed.2024.1502055

**Published:** 2025-01-17

**Authors:** Lung-Yi Mak, James Fung, Gladys Lo, Christine Shing-Yen Lo, Trevor Kwan-Hung Wu, Matthew Shing-Hin Chung, Tiffany Cho-Lam Wong, Wai-Kay Seto, Albert Chi-Yan Chan, Man-Fung Yuen

**Affiliations:** ^1^Department of Medicine, School of Clinical Medicine, The LKS Faculty of Medicine, The University of Hong Kong, Hong Kong SAR, China; ^2^State Key Laboratory of Liver Research, The LKS Faculty of Medicine, The University of Hong Kong, Hong Kong SAR, China; ^3^Department of Surgery, School of Clinical Medicine, The LKS Faculty of Medicine, The University of Hong Kong, Hong Kong SAR, China; ^4^Liver Transplantation Unit, Queen Mary Hospital, Hong Kong SAR, China; ^5^Department of Diagnostic and Interventional Radiology, Hong Kong Sanatorium and Hospital, Hong Kong SAR, China

**Keywords:** MASLD, liver transplant, metabolic dysfunction, implant biopsy, organ donation, steatotic graft

## Abstract

**Background:**

The rising prevalence of metabolic dysfunction-associated steatotic liver disease (MASLD) has led to an increased occurrence of steatotic liver grafts (SLG) in liver transplantation (LT). However, the implications of SLG on post-transplant *de novo* hepatic steatosis (PTHS) and advanced fibrosis (≥F3) remain uncertain. This study aimed to characterize PTHS and ≥ F3 using magnetic resonance imaging (MRI) in patients who underwent LT for non-MASLD indications and to examine their relationship with SLG.

**Methods:**

Post-LT patients with implant biopsy fat content data were recruited for MRI assessments. MRI-proton density fat fraction (MRI-PDFF) and MR elastography (MRE) were performed using a 1.5 Tesla Optima 450 W MR scanner with a 3D volumetric sequence. PTHS and ≥ F3 were defined as MRI-PDFF ≥5% and MRE ≥3.64 kPa, respectively. SLG was defined as implant biopsy fat content ≥5%.

**Results:**

A total of 292 patients (70.5% men, median age at LT: 51.9 years, 22.6% with SLG) were recruited. The majority (73.6%) were transplanted for hepatitis B virus (HBV)-related complications. MRI performed at a median of 12.2 years post-LT identified PTHS in 27.4 and 10.6% of patients. PTHS was independently associated with SLG (OR 2.067, 95% CI 1.082–3.951), central obesity (OR 3.952, 95% CI 1.768–8.832), and hypertension (OR 2.510, 95% CI 1.268–4.966). In contrast, ≥F3 was associated with sex, change in BMI, and abnormal liver biochemistry but not with PTHS or SLG.

**Conclusion:**

MRI identified a high prevalence of PTHS, which was associated with SLG and metabolic risk factors among Chinese patients transplanted for non-MASLD indications. Advanced graft fibrosis was not associated with PTHS or SLG.

## Introduction

Liver transplantation (LT) is often the only curative option for patients with acute liver failure, end-stage liver disease, and hepatocellular carcinoma (HCC). Post-LT, patients now achieve significantly improved survival rates, with an expected 10-year survival exceeding 80% (1).

Non-liver conditions, such as metabolic syndrome—comprising diabetes mellitus (DM), hypertension, and dyslipidemia—have emerged as important contributors to post-transplant morbidity and mortality. It is not surprising to note that metabolic syndrome has become a common emerging problem after LT, with reported rates of up to 50–60% (2). The metabolic syndrome is associated with the development of cardiovascular and cerebrovascular complications and is linked to the recurrence or development of *de novo* graft non-alcoholic fatty liver disease (NAFLD) (3), recently renamed metabolic-dysfunction-associated steatotic liver disease (MASLD) (4).

Previous studies have reported post-transplant MASLD recurrence rates ranging from 20 to 40% (5). The majority of the studies have relied on retrospective reviews of liver biopsies, with results limited to only those who required a liver biopsy for other reasons. Nevertheless, liver biopsy is not a feasible screening tool for all patients. Vibration-controlled transient elastography (VCTE) with a controlled attenuation parameter (CAP) has been shown to be a rapid, reliable, and repeatable non-invasive method for the assessment of liver steatosis, with high patient acceptance (6). This technique enables large-scale screening of liver transplant recipients for graft steatosis and fibrosis. Our group reported a high prevalence of post-transplant *de novo* hepatic steatosis (PTHS) in post-LT patients (28.9%), of which 95.6% fulfilled the criteria for MASLD, even though MASLD is still not a common indication for LT in Chinese people (7). Recently, magnetic resonance (MR) imaging (MRI) techniques have been widely used as a non-invasive modality to accurately assess hepatic fibrosis and steatosis. MRI proton density fat fraction (PDFF) can calculate liver fat content in an accurate, repeatable, and reproducible way (8–11).

Magnetic resonance elastography (MRE) offers three-dimensional measurements of liver stiffness (12, 13). Compared to VCTE, MRI-PDFF, and MRE are more accurate ways for fat quantification and fibrosis assessment, respectively (14, 15). Several risk factors have been identified in the recurrence of MASLD or PTHS, including obesity, metabolic syndrome, diabetes, dyslipidemia, and sirolimus exposure, while the role of donor characteristics and graft steatosis remains controversial (16–18).

We aimed to quantify the prevalence of PTHS in post-LT patients using MRI-PDFF and assess the prevalence of advanced fibrosis with MRE. We evaluated predictive factors for PTHS, advanced fibrosis, and their relationship with donor graft steatosis.

## Methods

### Study design

This cross-sectional study was conducted in the Department of Medicine and Surgery of Queen Mary Hospital and the Department of Diagnostic and Interventional Radiology of Hong Kong Sanatorium and Hospital, Hong Kong. Adult patients who underwent LT, were regularly followed up, and had a prior valid VCTE assessment, as described in a previous study (19) were screened for eligibility. Patients were excluded if there were contraindications to MR imaging, such as the presence of metallic implants (e.g., non-MRI-compatible pacemaker or cerebral aneurysm clips) and claustrophobia or had been re-transplanted after the first VCTE.

Clinical data, including age, gender, donor type (deceased donor vs. living donor), indication for LT, concomitant medications, and medical comorbidities, were recorded. The study protocol conformed to the ethical guidelines of the 1975 Declarations of Helsinki as reflected in *a priori* approval by the Institutional Review Board/Ethics Committee of the University of Hong Kong and the Hospital Authority Hong Kong West Cluster (reference number: UW 20–689). Written informed consent was obtained from each patient.

### Assessment

Between October 2020 and September 2023, eligible patients who provided valid written informed consent were recruited. All patients had implant biopsies at the time of transplant, and the fat percentage in the implant biopsy was recorded. Steatotic liver graft (SLG) was defined as a percentage fat content ≥5% on implant biopsy, with 5–32.9% classified as mild steatosis, 33–66% as moderate steatosis, and > 66% as severe steatosis, in accordance with the NASH Clinical Network Scoring System definitions (20, 21). Following recruitment, the anthropometric assessment was performed, including body mass index (BMI) and waist circumference. Patients underwent paired MRI-PDFF, MRE, and VCTE assessments. Fasting glucose and lipid profiles, as well as liver biochemistry, were assessed. Abnormal liver biochemistry was defined as a persistent elevation of alanine aminotransferase (ALT), aspartate aminotransferase (AST), alkaline phosphatase (ALP), gamma-glutamyl transferase (GGT), or bilirubin at least twice the upper limit of normal. Metabolic risk factors were defined as the presence of at least one of the following: (1) central obesity as shown by waist circumference ≥ 90/80 cm in Asian men and women, (2) high blood pressure ≥ 130/85, (3) hypertriglyceridemia ≥1.7 mmol/L or on lipid-lowering treatment, (4) reduced high-density lipoprotein (HDL) cholesterol <1.0 or 1.3 mmol/L for men or women or on lipid-lowering treatment, and (5) fasting glucose ≥5.6 mmol/L or on anti-diabetic treatment (22). For BMI, Asian-specific cut-off values were adopted as follows: underweight: BMI <18 kg/m^2^, normal: BMI 18–22.9 kg/m^2^, overweight: BMI 23–24.9 kg/m^2^, obese: BMI ≥25 kg/m^2^ (23).

### MRI-PDFF and MRE

MRE and MRI-PDFF were conducted using a 1.5 Tesla Optima 450 W MR scanner (General Electric, Fairfield, Connecticut). MR operators were blinded to the clinical data of all study participants. The scanning protocol included an MR-sequence software product (IDEAL-IQ; GE Healthcare), which is a three-dimensional volumetric imaging sequence acquired through a single breath-hold. The acquired images were sent to the GE Advantage Window to acquire fat measurements (expressed in percentages) and MR elastography values (expressed in kPa). The arithmetic mean values for fat percentage and elastography readings were calculated using all available data points. PTHS and ≥ F3 were defined as MRI-PDFF ≥5% and MRE ≥3.64 kPa, respectively (24, 25). The total scanning time was approximately 10–15 min per patient (14, 26).

### VCTE

All recruited patients underwent VCTE assessment using the M probe unless otherwise specified. Hepatic fat quantification was assessed by CAP, and a median value (expressed in dB/m) was obtained after ≥10 reliable acquisition, defined as an interquartile range (IQR) <40 dB/m. Liver fibrosis was measured by liver stiffness (LS), and a median value (expressed in kPa) was obtained after ≥10 reliable acquisitions, defined as ≥60% success rate and IQR <30%. Two fully trained and certified operators performed VTCE.

### Statistical analyses

The chi-squared test was used for categorical variables. Continuous variables were presented as median values, with IQR shown in brackets. Variables with skewed distribution were analyzed using the Mann–Whitney test. Those with three or more variables were analyzed using the Kruskal-Wallis test. Paired-related continuous variables were analyzed using the Wilcoxon paired test. Multivariate analysis was performed using bivariate logistic regression on significant univariate variables (defined as those with *p*-value <0.1). The correlation between two variables was assessed using the Pearson method. Subgroup analysis was performed among patients who were transplanted with a non-steatotic graft. All statistical analyses were performed using SPSS version 27.0 (IBM Corp., Armonk, NY). The Sankey diagram was constructed using Stata, version 17 (StataCorp LLC). A *p*-value of <0.05 was considered statistically significant.

## Results

### Cohort characteristics

A total of 292 patients (70.5% male, median age at LT: 51.9, IQR 45.9–56.8) were recruited. The majority of patients (73.6%) underwent transplantation due to hepatitis B virus (HBV)-related complications, which included acute HBV infection, acute flare of chronic hepatitis B, acute-on-chronic liver failure, decompensated cirrhosis, and HBV-related HCC.

Living donor liver transplantation (LDLT) was conducted in 53.8% of patients. The majority (77.4%) did not have excessive hepatic fat on implant biopsy, although one-quarter of patients received an SLG with varying degrees of steatosis at graft implantation. SLG was more common among deceased donor LT (DDLT) than LDLT (31.3% vs. 15.4%, *p* = 0.002). Diabetes mellitus, dyslipidemia, hypertension, and overweight were present in 11.3, 1.7, 9.9, and 32.9%, respectively, at the time of transplant (Table 1).

**Table 1:** Cohort characteristics of 292 patients at liver transplantation and at MRI.

Clinical parameter	Value (median or count)	Interquartile range or percentage
At liver transplantation		
Age (years)	51.9	45.9–56.8
Gender (being male)	206	70.5%
Indication of liver transplantation	HBV-related: 215 HCV: 15 Cryptogenic cirrhosis: 9 Autoimmune liver disease (PBC, AIH, or PSC): 18 Others: 35	73.6% 5.1% 3.1% 6.2% 12.0%
Type of donor	DDLT: 135 LDLT: 157	46.2% 53.8%
Implant biopsy fat	<5%: 226 5–32.9%: 48 33–66%: 10 >66%: 8	77.4% 16.4% 3.4% 2.7%
Body mass index (kg/m^2^)	21.3	18.9–23.9
Overweight/ obesity	96	32.9%
Diabetes mellitus	33	11.3%
Dyslipidaemia	5	1.7%
Hypertension	29	9.9%
At MRI assessment		
Age (years)	64.3	58.2–69.6
Time since transplant (years)	12.2	9.7–15.4
Diabetes mellitus	112	38.4%
Dyslipidaemia	66	22.6%
Hypertension	190	65.1%
Body mass index (kg/m^2^)	23.7	21.4–25.9
Overweight/ obesity	174	59.6%
Central obesity	178	61%
Immunosuppression		
tacrolimus	275	94.2%
rapamycin	24	8.2%
mycophenolate mofetil	66	22.6%
steroids	51	17.5%
Number of immunosuppressive drugs		
1	192	65.8%
≥2	100	34.2%
≥3	30	10.3%
Liver biochemistry		
Alanine aminotransferase (U/L)	22	16–30
Aspartate aminotransferase (U/L)	23	20–29
Alkaline phosphatase (U/L)	83	66–102
Gamma-glutamyl transferase (U/L)	31	20–64
Bilirubin (umol/L)	12	8–16
Abnormal liver biochemistry	57	19.5%

AIH, autoimmune hepatitis; DDLT, deceased donor liver transplantation; HBV, hepatitis B virus; HCV, hepatitis C virus; LDLT, living donor liver transplantation; MRI, magnetic resonance imaging; PBC, primary biliary cholangitis; PSC, primary sclerosing cholangitis.

MRI was performed at a median time of 12.2 years post-LT (IQR 9.7–15.4 years) when the median age of patients was 64.3 (IQR 58.2–69.6) years old. The median body mass index increased from 21.3 kg/m^2^ at LT to 23.7 kg/m^2^ at recruitment (*p* < 0.001), with 59.8% being overweight/obese (*p* < 0.001); the breakdown of BMI groups at both timepoints is shown in Supplementary Figure 2. The proportion of patients having diabetes mellitus, dyslipidemia, and hypertension increased to 38.4, 22.6, and 65.1%, respectively (all *p* < 0.001). The majority of patients (94.2%) were on tacrolimus, while others were on various immunosuppressants; most of them were receiving single-agent therapy (Table 1). Abnormal liver biochemistry was observed in 19.5% of patients.

### Prevalence of PTHS and ≥ F3 by MRI metrics

**Figure 1:**
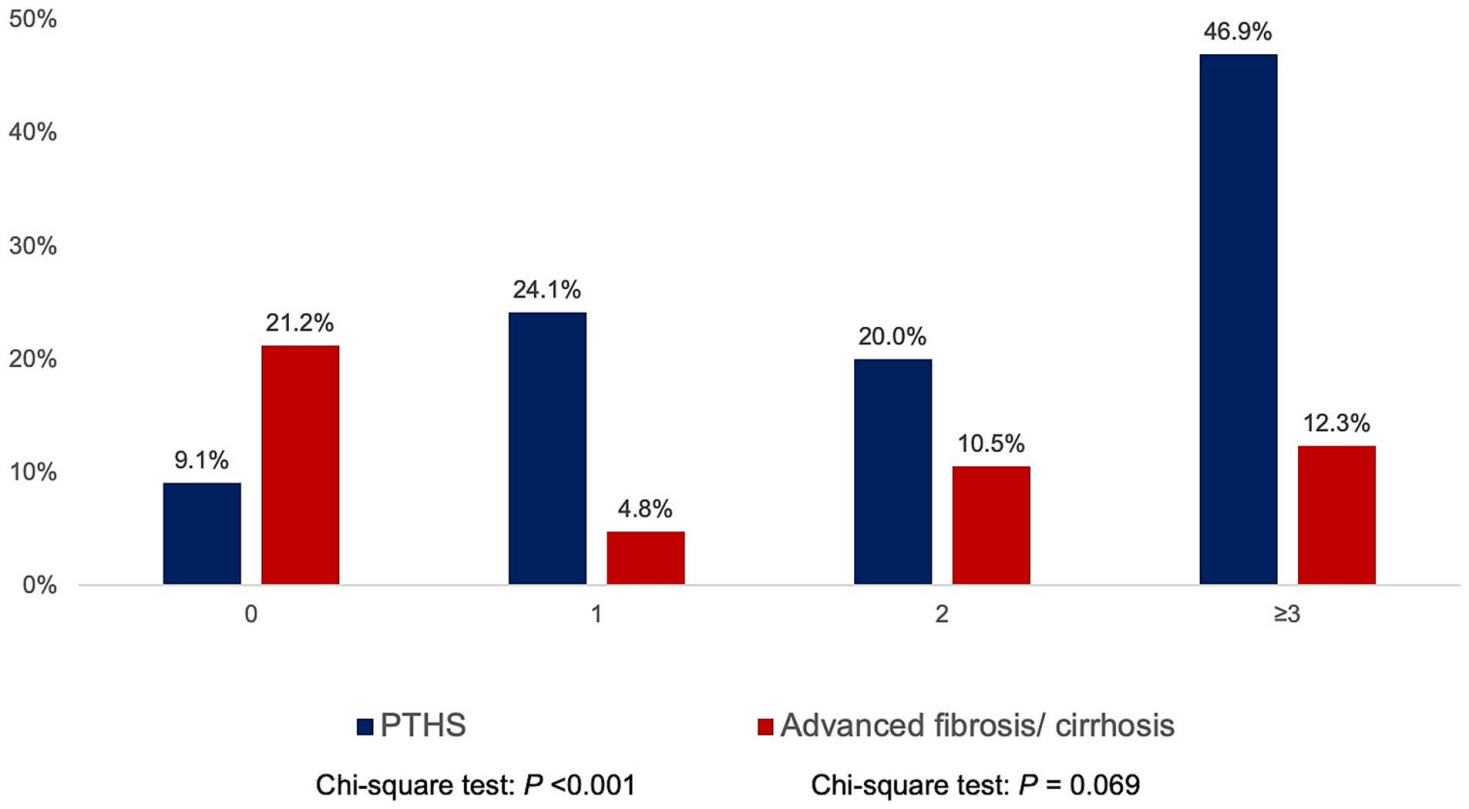
Prevalence of post-transplant *de novo* hepatic steatosis (PTHS) and advanced fibrosis according to the number of metabolic risk factors (hypertriglyceridemia, low high-density lipoprotein cholesterol, obesity, diabetes mellitus/ pre-diabetic, and hypertension).

The median MRI PDFF was 2.51% (IQR 1.66–5.34%), showing significant inter-segmental differences: S2 2.85% (IQR 1.23–5.02%), S3 2.29% (IQR 1.23–5.02%), S5 2.55% (IQR 1.29–5.30%), S6 2.67% (IQR 1.62–5.33%), S7 2.49% (IQR 1.42–4.99%), and S8 2.90% (IQR 1.73–5.81%). The median MRE was 2.43 kPa (IQR 2.18–2.91 kPa), with similar median values between the upper and lower segments. According to MRI criteria, PTHS and ≥ F3 were present in 27.4 and 10.6%, respectively. The prevalence of PTHS increased with higher BMI categories (Supplementary Figure 3). The prevalence of PTHS increased proportionally with the number of metabolic risk factors (0: 9.1%, 1: 24.1%, 2: 20%, ≥3: 46.9%, *p* < 0.001; Figure 1).

In comparison, the prevalence of ≥F3 did not demonstrate a consistent trend across BMI categories (Supplementary Figure 3) or metabolic risk factors (0: 21.2%, 1: 4.8%, 2: 10.5%, ≥3: 12.3%, *p* = 0.069; Figure 1). Patients who were transplanted for HBV-related complications had higher MRI-PDFF than non-HBV-LT (*p* = 0.023) but were not associated with significant differences in MRE (Supplementary Figure 1). Sex and donor type were not associated with significant differences in MRI-PDFF and MRE (all *p* > 0.05).

**Figure 2:**
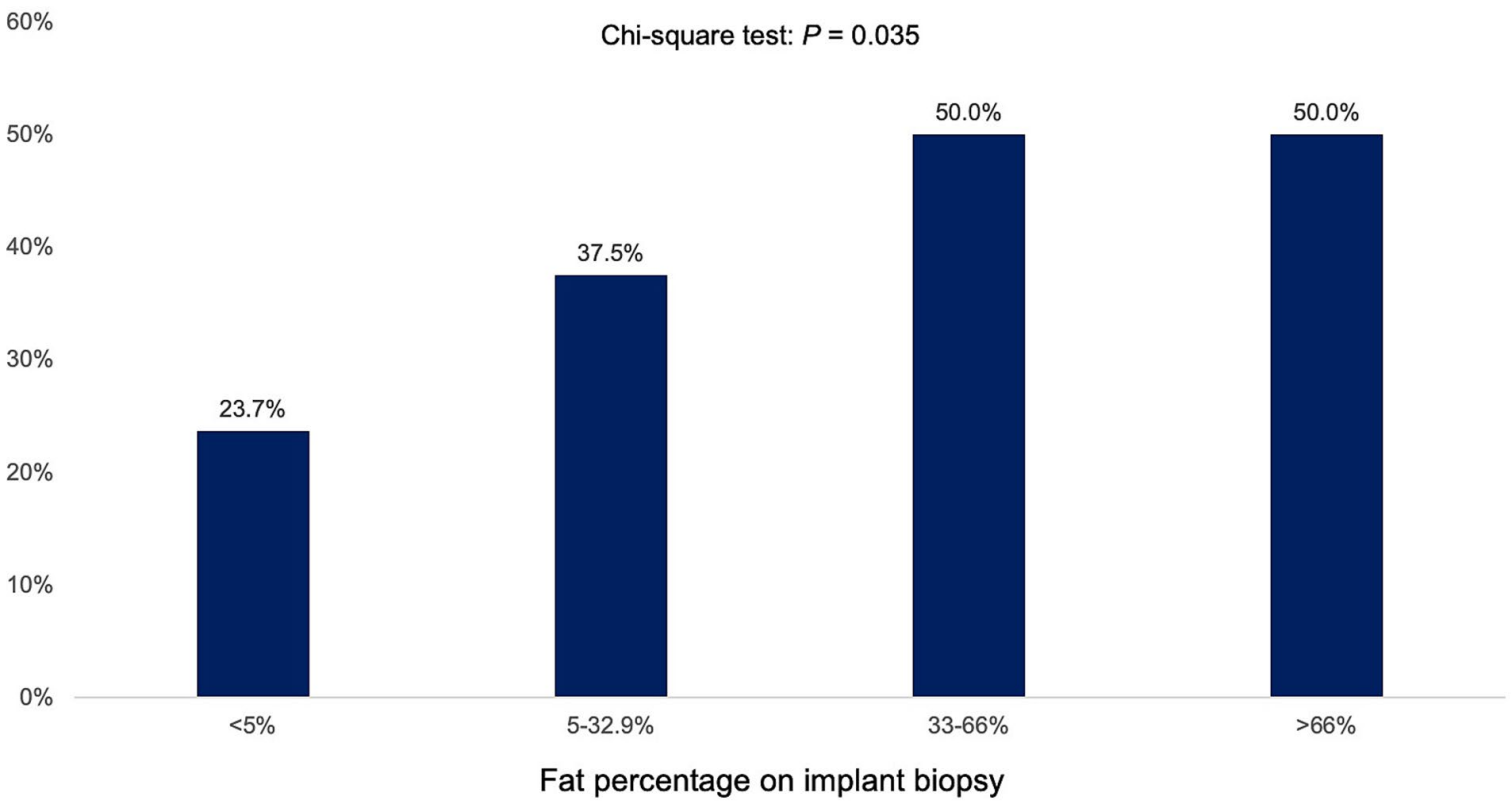
Prevalence of post-transplant *de novo* hepatic steatosis (PTHS) according to percentage of hepatic fat in implant biopsy.

Stratification by fat percentage on implant biopsy revealed that PTHS prevalence increased with the severity of graft steatosis (<5% implant biopsy fat: 23.7% PTHS, 5–32.9% implant biopsy fat: 37.5% PTHS, 33–66% implant biopsy fat: 50% PTHS, >66% implant biopsy fat: 50% PTHS, *p* = 0.035; Figure 2). However, the percentage of fat on implant biopsy was not associated with the prevalence of ≥F3 (<5% implant biopsy fat: 10.7% ≥ F3, 5–32.9% implant biopsy fat: 12.5% ≥ F3, 33–66% implant biopsy fat: 10% ≥ F3, >66% implant biopsy fat: 0% ≥ F3; *p* = 0.770).

### Predictors for PTHS and ≥ F3

**Figure 3:**
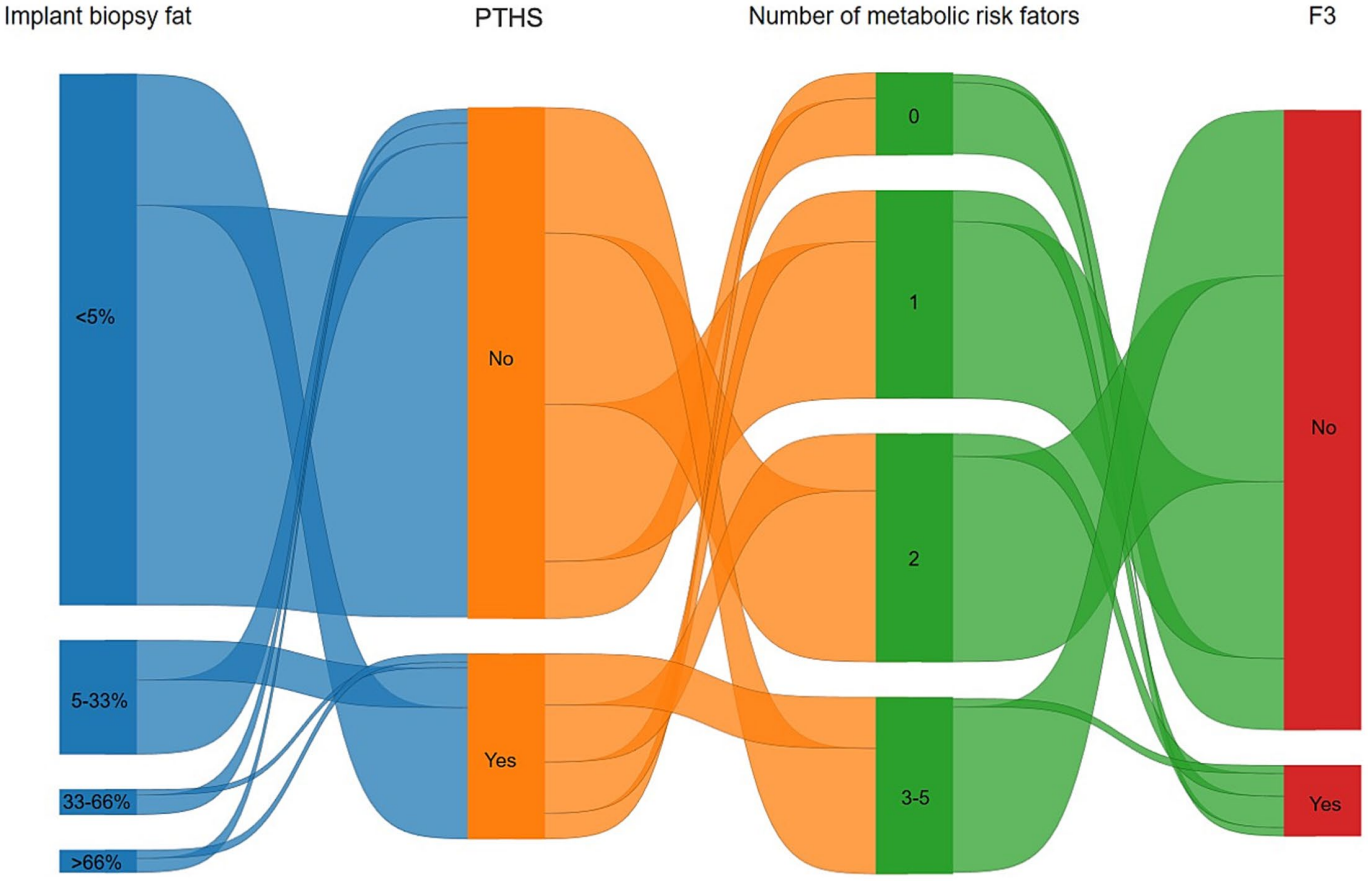
Sankey diagram highlighting the relationship between implant biopsy fat percentage, post-transplant *de novo* hepatic steatosis (PTHS), metabolic dysfunction, and at least advanced liver fibrosis (≥F3). PTHS occurs not only in patients with excessive hepatic fat on implant biopsy but also in those with minimal fat. The presence of advanced fibrosis was linked with a number of metabolic risk factors.

Figure 3 demonstrates the relationship between implant biopsy fat percentage, PTHS, metabolic dysfunction, and ≥ F3. PTHS occurs not only in patients who received SLG but also in those with minimal fat in the implant biopsy. The presence of ≥F3 was not significantly associated with metabolic risk factors.

Upon multivariate binary logistic regression analysis, SLG (OR 2.067, 95% CI 1.082–3.951, *p* = 0.028), central obesity (OR 3.952, 95% CI 1.768–8.832, *p* = 0.001), and hypertension (OR 2.510, 95% CI 1.268–4.966, *p* = 0.008) were independently associated with excessive hepatic steatosis after LT on MRI-PDFF (Table 2). Similar findings were obtained when the subgroup of patients who did not receive SLG was considered, confirming the association of central obesity (OR 2.630, 95% CI 1.095–6.321, *p* = 0.031) and hypertension (OR 2.362, 95% CI 1.059–5.267, *p* = 0.036) as independent risk factors for PTHS (Supplementary Table 1).

**Table 2:** Predictors for excessive hepatic steatosis after liver transplantation on MRI-PDFF.

	No	Yes	*p* value	Odds ratio	95% CI	*p* value
Age at LT	51.7	51.9	0.467			
Sex (male)	68.4%	76.3%	0.120			
HBV	71.2%	80%	0.084	1.042	0.511–2.126	0.910
LDLT	52.8%	56.3%	0.348			
Implant biopsy ≥5% fat (SLG)	18.6%	33.8%	0.005	2.067	1.082–3.951	0.028
Change in BMI	+2.2	+3.1	0.021	1.015	0.924–1.115	0.757
Central obesity	51.7%	86.3%	<0.001	3.952	1.768–8.832	0.001
overweight	50.7%	83.8%	<0.001	1.915	0.866–4.239	0.109
DM	39.3%	36.3%	0.365			
HT	58.8%	81.3%	<0.001	2.510	1.268–4.966	0.008
Dyslipidaemia	22.7%	22.5%	0.549			
≥F3	12.3%	6.3%	0.098	0.419	0.141–1.248	0.118
Tacrolimus use	93.9%	95%	0.806			
Rapamycin use	7.5%	10%	0.321			
MMF use	22.6%	22.5%	0.557			
Steroid	18.4%	15%	0.310			

≥F3, at least advanced fibrosis; CI, confidence interval; DM, diabetes mellitus; HBV, hepatitis B virus; HT, hypertension; LDLT, living donor liver transplantation; LT, liver transplantation; MMF, mycophenolate mofetil; MRI-PDFF, magnetic resonance imaging proton density fat fraction; SLG, steatotic liver graft (at implantation).

For ≥F3, male sex (OR 0.326, 95% CI 0.108–0.981, *p* = 0.046), change in BMI (OR 0.848, 95% CI 0.742–0.970, *p* = 0.016), ALT (OR 0.944, 95% CI 0.900–0.990, *p* = 0.017), AST (OR 1.109, 95% CI 1.046–1.177, *p* = 0.001), and GGT (OR 1.016, 95% CI 1.006–1.026, *p* = 0.001) were independent variables in multivariate binary logistic regression (Table 3). In comparison, age, indication of LT, use of SLG (even with graft implant biopsy ≥33% fat), ALP, and metabolic risk factors were not associated with PTHS or ≥ F3 (Table 3). Similar findings were obtained when the subgroup of patients who did not receive SLG was considered, confirming the association of ALT (OR 0.922, 95% CI 0.870–0.978, *p* = 0.007) and AST (OR 1.153, 95% CI 1.068–1.246, *p* < 0.001) as independent risk factors for ≥F3 (Supplementary Table 2).

**Table 3:** Predictors for at least advanced fibrosis after liver transplantation on MRE.

	No	Yes	*p* value	Odds ratio	95% CI	*p* value
Age at LT	51.8	52.3	0.749			
Sex (Male)	72.4%	54.8%	0.037	0.326	0.108–0.981	0.046
HBV	75.9%	54.8%	0.013	0.565	0.195–1.640	0.294
LDLT	52.9%	61.3%	0.243			
Implant biopsy ≥5% fat	22.8%	22.6%	0.592			
Implant biopsy ≥33% fat	6.6%	3.2%	0.404			
Change in BMI	+2.5	+1.4	0.024	0.848	0.742–0.970	0.016
Central obesity	61.2%	61.3%	0.576			
overweight	61.2%	48.4%	0.120			
DM	36.9%	51.6%	0.083	1.916	0.741–4.950	0.180
HT	63.5%	77.4%	0.087	2.633	0.836–8.297	0.098
Lipids	23.1%	19.4%	0.417			
PTHS	28.7%	16.1%	0.098	0.620	0.175–2.199	0.459
ALT	21	29	0.004	0.944	0.900–0.990	0.017
AST	23	31	<0.001	1.109	1.046–1.177	0.001
ALP	82	100	<0.001	0.995	0.987–1.003	0.200
GGT	27	58	<0.001	1.016	1.006–1.026	0.001
Bilirubin	12	13	0.218			
Tacrolimus use	93.5%	100%	0.342			
Rapamycin use	8.4%	6.5%	0.519			
MMF use	21.1%	35.5%	0.061	2.463	0.930–6.525	0.070
Steroid	17.6%	16.1%	0.534			

ALP, alkaline phosphatase; ALT, alanine aminotransferase; AST, aspartate aminotransferase; CI, confidence interval; DM, diabetes mellitus; GGT, gamma-glutamyl transferase; HBV, hepatitis B virus; HT, hypertension; LDLT, living donor liver transplantation; LT, liver transplantation; MMF, mycophenolate mofetil; MRI, magnetic resonance imaging; PTHS, post-transplant hepatic steatosis; SLG, steatotic liver graft (at implantation).

While steroids are known to have steatogenic effects, they were not associated with PTHS in our cohort. Upon sensitivity analysis by excluding long-term steroid users (*n* = 51), the prevalence of PTHS and ≥ F3 were 28.2 and 10.8%, respectively, which were similar to the main group results. Further analysis of the number of immunosuppressive drugs revealed no association with PTHS or ≥ F3.

### VCTE vs. MRI

Given the greater availability and broader applicability of VCTE compared to MRI, we examined the performance of VCTE in detecting PTHS and ≥ F3 using MRI metrics as the reference standard.

**Figure 4:**
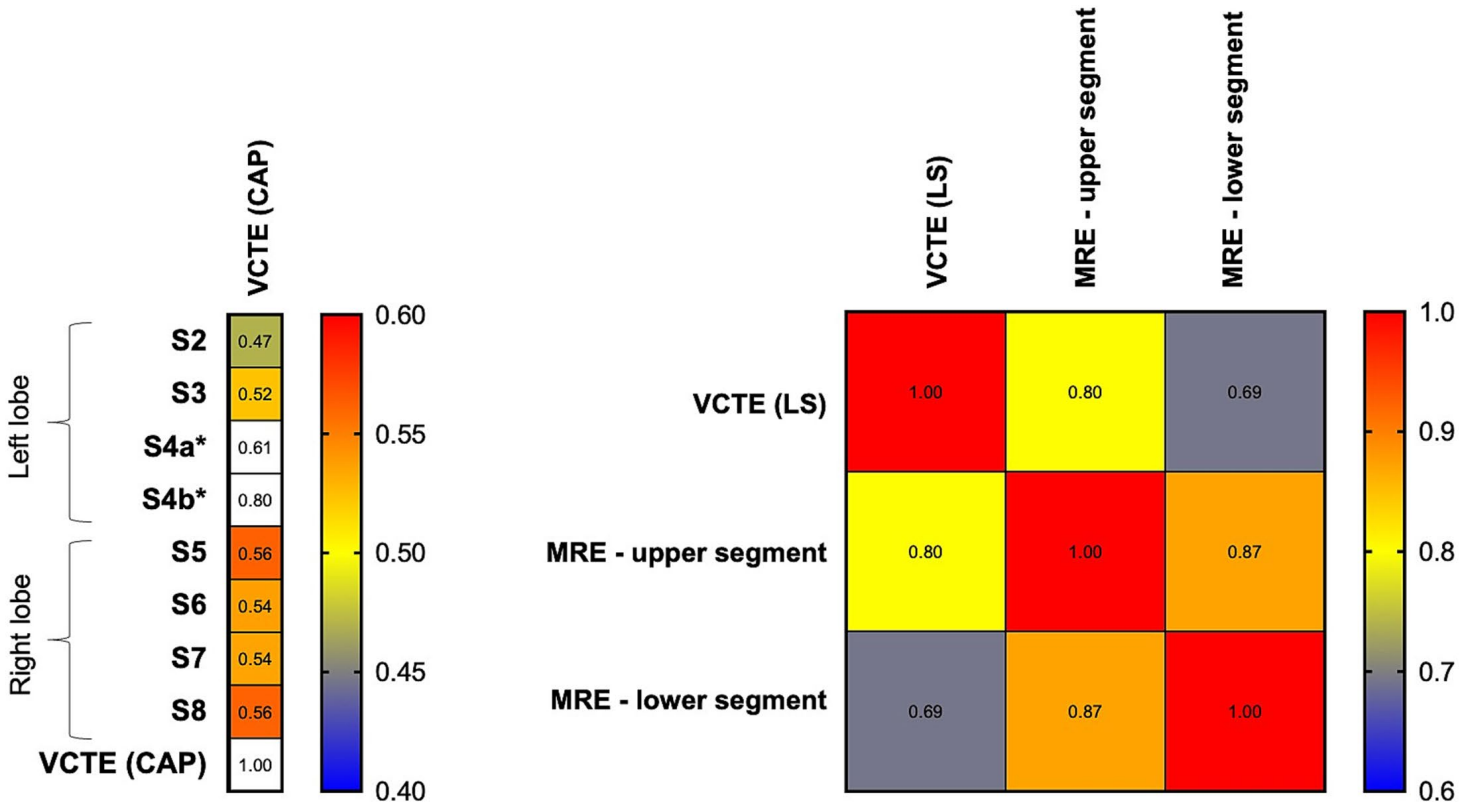
Heat map showing a correlation between MRI and VCTE parameters. Left panel: VCTE (CAP) demonstrated a higher correlation with right-sided hepatic segments (S5, S6, S7, S8) than left-sided segments (S2 and S3). *S4a and S4b: limited data on MRI-PDFF. Right panel: VCTE (LS) demonstrated a higher correlation with an upper segment of the liver than a lower segment of the liver. CAP, controlled attenuation parameter; LS, liver stiffness; MRE, magnetic resonance elastography; S2-S8: various hepatic segments on magnetic resonance imaging proton density fat fraction, and VCTE, vibration-controlled transient elastography.

VCTE-CAP moderately correlated with MRI-PDFF (CAP: *r* = 0.61, *p* < 0.001). A breakdown of MRI-PDFF demonstrated a better correlation between VCTE and the right lobe (S5: 0.56, S6: 0.54, S7: 0.54, S8: 0.56) than the left lobe (S2: 0.47, S3: 0.52), all *p* < 0.001 (Figure 4). The area under the receiver-operating characteristic curve (AUROC) for VCTE-CAP in identifying hepatic steatosis was 0.812 (95% CI 0.757–0.868, *p* < 0.001). By maximizing Youden’s index, a cutoff value of 246 dB/m on VCTE was found to have a sensitivity of 78.5% and a specificity of 70% for identifying hepatic steatosis on MRI-PDFF.

VCTE-LS is highly correlated with MRE at recruitment (*r* = 0.77, *p* < 0.001). A breakdown of MRE demonstrated a better correlation between VCTE and the upper segment (*r* = 0.80) than the lower segment (*r* = 0.69); both *p* < 0.001 (Figure 4). The AUROC for VCTE-LS in identifying ≥F3 was 0.890 (95% CI 0.820–0.960, *p* < 0.001). By maximizing Youden’s index, a cutoff value of 7.75 kPa on VCTE achieved 83.3% sensitivity and 81.6% specificity for identifying ≥F3 on MRE.

## Discussion

In this long-term study involving 292 Chinese patients who received LT for non-MASLD indications almost a decade ago, MRI-PDFF revealed a high prevalence of PTHS, affecting one-quarter of the cohort. These findings are coherent with existing literature on post-LT MASLD recurrence and *de novo* hepatic steatosis (17, 27), and indeed similar to the background prevalence rate of MASLD in the general population (28). In comparison, the prevalence of at least advanced fibrosis (≥F3) was 10.6%, which was not exceptionally high given the high prevalence of PTHS, the relatively advanced age of the cohort at recruitment (median age: 64.3 years), and the long post-transplant duration. Our findings are also coherent with conclusions drawn from other studies, which suggested the lack of association between post-LT MASLD and long-term clinical outcomes, including overall mortality (16, 29).

We identified SLG, central obesity, and hypertension to be independent risk factors for PTHS. Notably, PTHS can develop even in the absence of SLG, as shown in Figure 3. SLG was used in about one-quarter of LTs in this cohort, with more SLG from deceased donors than from living donors, which is concordant with the strict donor selection criteria when LDLT was contemplated. The liberal use of LDLT in Hong Kong and many other countries and regions in the Asia Pacific is distinct from the situation in Europe and North America (30), where most liver grafts are from deceased donors and less stringent graft selection criteria are to be followed. These livers are often regarded as extended criteria or marginal livers, but the post-LT outcomes have been shown to be not directly related to SLG (31). Therefore, the use of SLG is becoming more liberal in the West (32). Our study shows that the use of SLG and PTHS *per se* was not associated with ≥F3 in the post-LT setting. These findings are especially relevant to most regions in the world with limited availability of liver grafts, as the threshold to accept SLG can be further lowered to allow more grafts to be used due to the favorable prognosis even with graft steatosis at implant and development of PTHS after LT. Furthermore, preliminary data suggest that using machine perfusion for SLG prior to LT results in similar post-transplant mortality, severe complications, and peak ALT levels compared to non-SLGs (33), which might further broaden the use of SLG but requires further validation.

We noted that metabolic dysfunction was not associated with ≥F3. Instead, male sex and change in BMI were negatively associated with ≥F3 after LT. The reasons for these observations were not clear. In addition, abnormal liver biochemistry (low ALT, high AST, high GGT) was positively associated with ≥F3 after LT. Abnormal liver biochemistry could be secondary to recurrence of the underlying disease leading to LT, e.g., autoimmune disease/recurrent HCV; chronic complications of LT, e.g., vascular complications; or chronic rejection of the liver graft. It is important to control these conditions to prevent the progression of liver fibrosis. The reason for the disparate findings of ALT and AST with regards to ≥F3 was unclear but could be explained by the phenomenon of AST: ALT ratio reversal observed in advanced liver disease.

Patients transplanted for HBV-related complications had higher MRI-PDFF than those transplanted for other indications.

At the time of LT and MRI, the median BMI of patients transplanted for HBV-related indications was significantly higher than that of patients transplanted for other indications (at LT: 21.5 kg/m^2^ vs. 20.6 kg/m^2^, *p* = 0.003; at MRI: 24.1 kg/m^2^ vs. 21.9 kg/m^2^, *p* < 0.001). Among patients with HBV, 65 out of 215 (30.2%) were transplanted due to HBV-related HCC, while the remaining patients were transplanted for acute-on-chronic liver failure (ACLF) or decompensated cirrhosis. As patients with HBV can develop HCC in a non-cirrhotic liver (34, 35), patients transplanted for HCC may retain better-preserved hepatic function compared to those transplanted for other etiologies, e.g., decompensated cirrhosis, acute liver failure, or ACLF. This could lead to more preserved body mass and a higher BMI for the group with HBV-related complications, leading to the higher observed MRI-PDFF. However, HBV-related transplant was not a risk factor for PTHS (Table 2).

Our study utilized MRI as the reference standard and VCTE as a surrogate tool to assess hepatic fat content and liver fibrosis in the post-LT setting. The two modalities showed a good correlation with the high accuracy of VCTE in diagnosing PTHS and ≥ F3. MRI assessment is the most accurate non-invasive test for liver fat and fibrosis (36). The advantage of MRI over VCTE is the possibility of selecting the region of interest so that regions prone to artifact, such as those near surgical clips or major vessels, can be avoided.

In addition, due to the known heterogeneous or focal involvement of fat within the liver (37), MRI assessments provide multiple data points across different segments, helping to minimize sampling bias. We confirmed the inter-segmental variability of MRI-PDFF readings and the resultant differences in correlation with VCTE-CAP (Figure 4). VCTE is more accurate for the right hepatic lobe (for CAP) and upper segment measurement (for LS), likely because the point of skin contact of the VCTE probe is located exactly where the right upper segments of the liver are. The clinical relevance of these inter-segmental variations and the potential of performing VCTE at ≥1 point of skin contact remain to be explored. Nevertheless, VCTE has excellent performance characteristics for PTHS and advanced fibrosis when using MRI as the ground truth (AUROC 0.812 and 0.890, respectively). Considering the availability of non-invasive tests and cost, the current study supports the routine assessment by VCTE to identify PTHS and graft advanced fibrosis in the post-LT setting.

Our study is limited by the lack of post-LT liver biopsies to validate the MRI and VCTE findings. However, MRI-PDFF is currently regarded as the most accurate non-invasive imaging to evaluate hepatic fat and is widely accepted as the gold standard (25).

Due to the retrospective nature of this study, other confounding factors contributing to hepatic insult and graft fibrosis could not be excluded. Additionally, the long interval between LT and MRI resulted in some patients being lost to follow-up for various reasons, preventing the assessment of their post-LT outcomes (38). Furthermore, SLG is known to be associated with primary graft non-function and poor post-LT outcomes, including graft loss, re-transplantation, or even death. As a result, the current cohort represents hepatic outcomes from long-term survivors, and the possibility of selection bias cannot be ruled out.

In conclusion, MRI revealed a high prevalence of PTHS associated with SLG and metabolic risk factors among Chinese patients transplanted for non-MASLD indications. Using MRI metrics as the reference standard, VCTE proved to be a reliable tool for identifying PTHS and ≥ F3 in the post-LT setting. Advanced graft fibrosis was not associated with SLG, PTHS, or donor type but was associated with sex, changes in BMI, and abnormal liver biochemistry. These findings suggest that the threshold for using SLG could potentially be lowered, considering the ongoing organ shortage.

## Data Availability

The original contributions presented in the study are included in the article/Supplementary material, further inquiries can be directed to the corresponding authors.
