# Supplementary material for: Impact of liver graft steatosis on long-term post-transplant hepatic steatosis and fibrosis via magnetic resonance quantification
Source: Front Med (Lausanne). 2025 Jan 17;11:1502055. doi: 10.3389/fmed.2024.1502055 (PMC11782125; doi:10.3389/fmed.2024.1502055)
Supplement: Supplementary file 1 [file Presentation_1.pdf]

The impact of liver graft steatosis on long-term post-transplant hepatic steatosis and fibrosis via magnetic resonance quantification

Lung-Yi Mak, James Fung, Gladys Lo, Christine Shing-Yen Lo, Trevor Kwan-Hung Wu, Matthew Shing-Hin Chung, Tiffany Cho-Lam Wong, Wai-Kay Seto, Albert Chi-Yan Chan, Man-Fung Yuen

Supplementary Material

Supplementary Figure 1. MRI parameters stratified by indication of LT: HBV-related vs non-HBV related

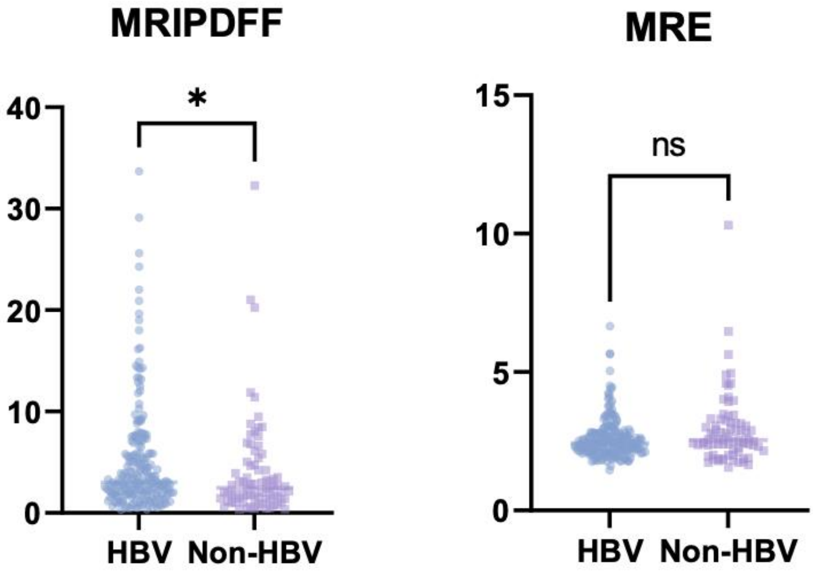

Supplementary Table 1. Predictors for excessive hepatic steatosis after liver transplantation on MRI-PDFF among patients who received non-steatotic liver graft (n=226)

	**No**	**Yes**	**P value**	**Odds ratio**	**95% CI**	**P value**
Age at LT	52.3	51.8	0.927			
Gender (male)	67.3%	75.5%	0.168			
HBV	69%	84.9%	0.015	1.671	0.689-4.050	0.256
LDLT	57.9%	62.3%	0.633			
Central obesity	53.2%	83%	<0.001	2.630	1.095-6.321	0.031
overweight	49.4%	81.1%	<0.001	2.324	0.988-5.467	0.053
DM	40.6%	34%	0.423			
HT	57.6%	81.1%	0.002	2.362	1.059-5.267	0.036
Dyslipidaemia	22.4%	17%	0.447			
≥F3	12.3%	5.7%	0.211			
Tacrolimus use	93.6%	94.3%	0.854			
Rapamycin use	7.6%	7.5%	0.627			
MMF use	24%	17%	0.347			
Steroid	17.5%	13.2%	0.531			

≥F3: at least advanced fibrosis, CI: confidence interval, DM: diabetes mellitus, HBV: hepatitis B virus, HT: hypertension, LDLT: living donor liver transplantation, LT: liver transplantation, MMF: mycophenolate mofetil, MRI-PDFF: magnetic resonance imaging proton density fat fraction, SLG: steatotic liver graft (at implantation)

Supplementary Table 2. Predictors for at least advanced fibrosis after liver transplantation on MRE among patients who received non-steatotic liver graft (n=226)

	**No**	**Yes**	**P value**	**Odds ratio**	**95% CI**	**P value**
Age at LT	52.1	52.3	0.847			
Gender (Male)	71%	54.2%	0.104			
HBV	74.5%	58.3%	0.143			
LDLT	58.5%	62.5%	0.827			
Central obesity	60%	62.5%	0.499			
overweight	57.8%	50%	0.517			
DM	38.2%	45.8%	0.510			
HT	61.8%	75%	0.265			
Lipids	21.6%	16.7%	0.792			
PTHS	25%	12.5%	0.211			
ALT	22	27	0.031	0.922	0.870-0.978	0.007
AST	23	30	<0.001	1.153	1.068-1.246	<0.001
ALP	83	92	0.024	1.000	0.992-1.008	0.963
GGT	27	54	<0.001	1.007	0.999-1.016	0.099
Bilirubin	12	14	0.177			
Tacrolimus use	93%	100%	0.408			
Rapamycin use	8%	4.2%	0.434			
MMF use	21.5%	29.2%	0.269			
Steroid	17%	12.5%	0.413			

ALP: alkaline phosphatase, ALT: alanine aminotransferase, AST: aspartate aminotransferase, CI: confidence interval, DM: diabetes mellitus, GGT: gamma glutamyl transferase, HBV: hepatitis B virus, HT: hypertension, LDLT: living donor liver transplantation, LT: liver transplantation, MMF: mycophenolate mofetil, MRI: magnetic resonance imaging, PTHS: post-transplant hepatic steatosis, SLG: steatotic liver graft (at implantation)
